# Supplementary material for: Patterns of Intron Gain and Loss in Fungi
Source: PLoS Biol. 2004 Nov 30;2(12):e422. doi: 10.1371/journal.pbio.0020422 (PMC532390; doi:10.1371/journal.pbio.0020422)
Supplement: Table S1 — Also available at http://genes.mit.edu/NielsenEtAl/. (4.3 MB ZIP). [file pbio.0020422.st001.zip › NielsenEtAl/html/355.html]

AN1188.1.NCU02633.1.MG04442.1.FG01010.1


```
 CLUSTAL W (1.82) Multiple Sequence Alignments - Introns Inserted


Sequence 1: NCU02633.1	432 aa
Sequence 2: FG01010.1	376 aa
Sequence 3: MG04442.1	410 aa
Sequence 4: AN1188.1	502 aa
Alignment Length: 581 aa
Number Identitical Residues: 143 aa
Alignment Score (without introns) 7249


MG04442.1 	-MAEPQQHDDQPQEPVAEFWSFPTDPAEFDNDERVSFSRLENKYIAVQEDGAEYEWDGGL
NCU02633.1	--------MEQQEGQQAEYWTFPTDPGEFDKDERISFSKLDNKYIAVQDDGTEYEFDEGL
FG01010.1 	--------MDLP---------FPIDPREFDSDDRISFSKLDKKFIAVHDDGNEYEFDADS
AN1188.1  	MALQDPKHDPTATGSPPAISNFPQDPSDFDSDPRVSFSKLDNKFILETDDGQEYSYDTIL
          	   .  .         .   .** ** :**.* *:***:*::*:*   :** **.:*   

MG04442.1 	KRWIPI~VDDALIESHQAAYGGGAAEDEEAPATTAADRRKKKRKAGDD~D~G-~------
NCU02633.1	KRWLPI~IDEALIEEHQRAYITSLGDDDTETGQLGKGQGKKRKNDDRE0D~SA~SSNHNM
FG01010.1 	KRWILA~DEEPLEPPVTDVYDDFAEFASNEAAEDG---SRKRKNGSGQ~D~SE0TPEP--
AN1188.1  	KRWIPT0IDDDLLRQQQEAYKVEGVDEDEQAGAQR---LKKKRKQGSE~E0GD~------
          	***:    :: *      .*       .  ..       :*::: . : : .        

MG04442.1 	----------GKAKR-PRQNTAVYVTGLPLDVTVEEIAELFSRKCGVIAENIDDSSPRIK
NCU02633.1	NARPSKNRKREKAPREPKQNTAVYVTGLPLDATADEVAELFSRKCGVIAEEIDSGRPRIK
FG01010.1 	-SKPSRPNKKQKAPPQPKQNTAVYVTGLPPDTTVEEVHDLFSRKGGVIAEEIDSGAPRIK
AN1188.1  	----------AQKPKKQRVNTAVFVTSIPLDAEFDEIRDIFSR-CGVIAEEIDSGRPRIK
          	           :   . : ****:**.:* *.  :*: ::***  *****:**.. ****

MG04442.1 	LYTDGGGNFKGDALVVFFKPQSVDMAIMLLDDTDFRITPSGLSSGRIRVQAADTSYKRNN
NCU02633.1	MYTDANGNFKGDALIVFFKPQSVEMAIMLLDDTDFRFEPAPDGS-RMRVQAADMSYKKTK
FG01010.1 	LYNDADGNFKGDALIVFFKPQSVEMAIMLLDDTDFRITASGTHEGRIKVQAADSSYKKVK
AN1188.1  	MYTDDEGKFKGEALVVYFRPESVNLAIQMLDDSDFRLGVPGPQG-PMRVQPADFSFKS--
          	:*.*  *:***:**:*:*:*:**::** :***:***:  .      ::**.** *:*   

MG04442.1 	YDQEGGAEGVDGAGSGNGNSGRGSAAPAAE--------------NGNGGHTRNTTDKAKV
NCU02633.1	YDAAAGAENGGGDKAGNGGAEGEGSSSSTPYNNITNPNPVSSDGGGNGHNQRSQQDKAKI
FG01010.1 	YDQEPGA---GGEKS-NGAAE---------------------------RKPQNNRDRQKI
AN1188.1  	QQEAP--------------------------------------------TKTSMRDKKKI
          	 :                                                  .  *: *:

MG04442.1 	IKKAQKMSA~KLADWDDDDPYPYLAAAVGAGAGGNGPTNKKAERTVILRHMFTLKELD~E
NCU02633.1	IKKTQKLSA~KLADWDDDEPS---PSARLLGPAIKEAKGGKWDKVVILRHMFTLEELN~E
FG01010.1 	IKKTQKLDA~KLADWDDDLPY---PGQ--------PEAATKWDKLVILRHMFTLEELE~E
AN1188.1  	IKRTQKLNN2KLADWDDDEPA------------ALVDTNSKFEKIVILKHMFTLQELE0E
          	**::**:.  ******** *                    * :: ***:*****:**: *

MG04442.1 	DPAALLEIKEDIRDECEKLGEVTNCVLFDLEEDGIVSVRFRTPDAAEACIK~LMHGRSFD
NCU02633.1	DPAALLEIKDDIREECAKLGPVTNVVLFDEEEEGIVSVKFATVEAAEACVH~LMHGRAFD
FG01010.1 	DPAALLEIKEDIREECAKLGTVTNVVLFDQEPEGIVSVKFKDTASAHACIN~LMHGRRFD
AN1188.1  	DPAAILDIKEDIREECSKLGEVTNVVLYDKEEAGVVSVRFSNPESARACVK0LMNGRFFG
          	****:*:**:***:** *** *** **:* *  *:***:*    :*.**:: **:** *.

MG04442.1 	GRTVQASLATGKEKYRRSKDK--------DDGLDEDDA------------------~---
NCU02633.1	GRIVEAFFATGKERFRKSKNK--------DAGEEDGGDD-----------------~---
FG01010.1 	GRTVEAFLATGKEKFNKSKDE--------ENHEDSD--------------------~---
AN1188.1  	GTAVEAYISDGSERFRKSNEKRAALEDLAEKGLDAEDDDENQRLDEFGTWLESSHT0TPL
          	*  *:* :: *.*::.:*::: :: .. ::   :  . ....  .. .:  .:: : :. 

MG04442.1 	--------------------------~--------------~------~----------~
NCU02633.1	--------------------------~--------------~------~----------~
FG01010.1 	--------------------------~--------------~------~----------~
AN1188.1  	QAMPLPLSNFEIMHMIRGISSRKLER1YGIMIAIGAFHDLP0YIPNDI1PLLAKKFYVE0
          	.: . . :. .      . :: . .   .   : .:  . .   ...  .  :..   . 

MG04442.1 	--------------------~---------------------------------
NCU02633.1	--------------------~---------------------------------
FG01010.1 	--------------------~---------------------------------
AN1188.1  	LSPPKTSQPQNPGKSTEPDC1SELSIEEASSSRKRDGNVRMEYIVTQTPNRPGD
          	 :...::.......::.... :. : ..:::: . ...   .   :.:.. ...
```
